# Combined analysis of the metabolome and transcriptome reveals the metabolic characteristics and candidate genes involved in alkaloid metabolism in *Heuchera micrantha* Douglas ex Lindl

**DOI:** 10.1186/s12870-024-05363-3

**Published:** 2024-07-06

**Authors:** Weichang Gong, Lina Xiong, Hongbo Fu

**Affiliations:** 1 School of Life Science, Qufu Normal University, Qufu, 273165 China; 2 Key Laboratory for Research and Utilization of Characteristic Biological Resources in Southern Yunnan, College of Biological and Agricultural Sciences, Honghe University, Mengzi, 661199 China

**Keywords:** *Heuchera micrantha*, Alkaloids, Metabolome, Transcriptome, Principal component analysis

## Abstract

**Background:**

Alkaloids, important secondary metabolites produced by plants, play a crucial role in responding to environmental stress. *Heuchera micrantha*, a well-known plant used in landscaping, has the ability to purify air, and absorb toxic and radioactive substances, showing strong environmental adaptability. However, there is still limited understanding of the accumulation characteristics and metabolic mechanism of alkaloids in *H. micrantha*.

**Results:**

In this study, four distinct varieties of *H. micrantha* were used to investigate the accumulation and metabolic traits of alkaloids in its leaves. We conducted a combined analysis of the plant’s metabolome and transcriptome. Our analysis identified 44 alkaloids metabolites in the leaves of the four *H. micrantha* varieties, with 26 showing different levels of accumulation among the groups. The HT and JQ varieties exhibited higher accumulation of differential alkaloid metabolites compared to YH and HY. We annotated the differential alkaloid metabolites to 22 metabolic pathways, including several alkaloid metabolism. Transcriptome data revealed 5064 differentially expressed genes involved in these metabolic pathways. Multivariate analysis showed that four key metabolites (N-hydroxytryptamine, L-tyramine, tryptamine, and 2-phenylethylamine) and three candidate genes (*Cluster-15488.116815*, *Cluster-15488.146268*, and *Cluster-15488.173297*) that merit further investigation.

**Conclusions:**

This study provided preliminarily insight into the molecular mechanism of the biosynthesis of alkaloids in *H. micrantha*. However, further analysis is required to elucidate the specific regulatory mechanisms of the candidate gene involved in the synthesis of key alkaloid metabolites. In summary, our findings provide important information about how alkaloid metabolites build up and the metabolic pathways involved in *H. micrantha* varieties. This gives us a good starting point for future research on the regulation mechanism, and development, and utilization of alkaloids in *H. micrantha*.

**Supplementary Information:**

The online version contains supplementary material available at 10.1186/s12870-024-05363-3.

## Background

*Heuchera micrantha*, commonly referred to as coral bell, belongs to the Saxifragaceae family and exhibits perennial herbaceous characteristics [1]. As the domestic flower industry has growm continuously, *H. micrantha* has recently been introduced to China [2]. *H. micrantha* demonstrstes strong environmental adaptability, being able to withstand lower temperatures, exhibiting strong cold resistance, and thriving in dark environments [3]. As an important landscaping plant, *H. micrantha* possesses the attributes of purifying air, absorbing toxic and radioactive substances, and enhancing the natural environment of its surroundings [2]. Additionally, *H. micrantha* is capable of enhancing its photosynthesis efficiency based on the light intensity it received, thereby augmenting the oxygen concentration and humidity of the surrounding air [4]. *H. micrantha* boasts significant ecological and economic value.

As people’s living standards improved, *H. micrantha* is becoming increasing well-known and widely utilized. At present, both introduced and newly bred varieties of *H. micrantha* are widely utilized in the market [5]. Limited research on environmental adaptability and physiological characteristics of *H. micrantha* restricts the introduction of diverse varieties, hindering the breeding of new strains. Additionally, the domestic research on the resistance of *H. micrantha* and the direction of the variety is not comprehensive. Therefore, the domestic domestication and breeding of resistant and high-quality *H. micrantha* varieties remain a crucial research focus [6]. Geographically speaking, China boasts a vast landmass and a substantial flower market, enabling adaptation to diverse environments and climates across its northern and southern regions. Residents of both the north and south can cultivate and enjoy diverse varieties of *H. micrantha* to cater to market demand [1]. Currently, innovative experiments and researches on *H. micrantha* primarily focus on its valuable enhancement, breeding of new varieties, resistance and adaptability studies, tissue culture, rapid propagation method, physiological characteristics and application development direction of *H. micrantha*. In winter in Guizhou, China, a comparison was made among the cold tolerance adaptability of seven varieties of *H. micrantha* [1]. The findings indicated that the primary factor determining the variance in cold tolerance was the specific variety. Among these varieties, ‘Hailang’ and ‘Plum Pudding’ exhibiting more peonounced adaptability. The most intuitive expression of the plants’ growth and health status was the comparison among them [7]. Three varieties were selected for a study examing the photosynthetic characteristics of *H. micrantha* during summer. The results showed that the variety ‘Paris’ of *H. micrantha* could rapidly adapt to the challenging environment of intense sunlight and high temperatures during summer. For instance, in the experiments conducted in southern China and other places, even in the summer outdoor green space cultivation, its adaptability remains exceptionally strong. Moreover, during the peak midday of sunlight intensity in summer, the photosynthesis of *H. micrantha* will stop obviously. The underling cause of this phenomenon is the decrease of carboxylase activity with the mesophyll cells of *H. micrantha*, triggered by excessively high temperatures. This is opposed to the stomatal closure observed on the leaves of *H. micrantha* due to the high ambient temperature, which makes CO_2_ enter the leaves [8].

Alkaloids typically exhibit a ring-shaped structure, optical activity, and significant physiological and pharmacological impacts [9]. They serve as an effective component in numerous traditional Chinese herbal medicines. Since ancient times, human beings have been using various natural herbs to treat diseases. In the context of modern biomedicine, the efficacy of these herbs can be attributed to their active ingredients, with alkaloids being among the most biologically valuable compounds [10]. Alkaloids, a subclass of nitrogenous alkaline organic compounds, primarily reside in plants and constitute approximately 20% of plant secondary metabolites, as reported by Tang et al. (2023) [11]. Alkaloids are ubiquitous in multiple plant families, including Ranunculaceae, Tetrandraceae, Papaveraceae, Magnoliaceae, Rutaceae, Lithospermaceae, Compositae, Leguminosae, and Lauraceae.

Alkaloids are compounds that occur naturally. Moreover, a primary reason to the biosynthesis of alkaloids lies in the utilization of their bitter or toxic nature for plant self-defense against external agents, particularly insects and herbivores. Contemporary research increasingly demonstrates that the majority of alkaloids possess potent toxic properties, as documented by Zan et al. (2022) [12]. Steroidal alkaloids and pyrrolizidine alkaloids have anti-microbial, anti-insect and anti-fungal effects, which can help plants resist the invasion of external harmful substances, and play an important role in maintaining the co-evolution between populations [13]. In the present study, four distinct varieties of *H. micrantha* were selected as the experimental materials. The alkaloid metabolites of these varieties were characterized and compared to elucidate differences. Additionally, transcriptome sequencing was conducted to identify molecular differences in alkaloid metabolites biosynthesis among varieties. This provided an valuable reference for further breeding programs of *H. micrantha*.

## Results

### Identification of alkaloid metabolites

**Fig. 1:**
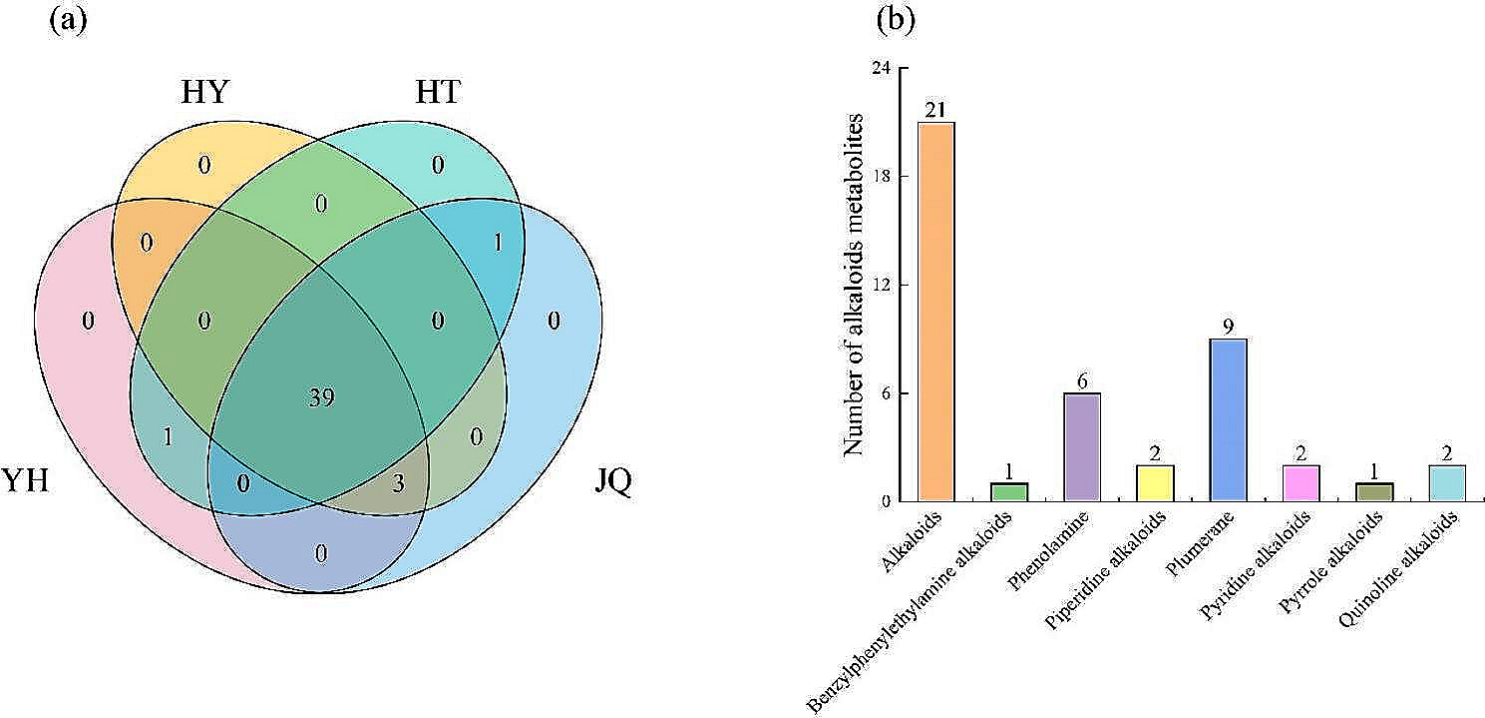
Venn diagram of alkaloid metabolites in each of the four *H. micrantha* varieties (**a**). The total numbers of alkaloid metabolites in different classes (**b**) extracted from four different *H. micrantha* varieties. YH, Yongheng; HY, Hongyue; HT, Huatan; JQ, Jinqiu

A total of 44 alkaloid metabolites were identified in the leaves of four varieties (YH, HY, HT, and JQ) of *H. micrantha* through UPLC-MS/MS analysis (Table S1). A total of 43 alkaloid metabolites were detected both in YH and JQ, 42 in HY, and 41 in HT. Thirty-nine alkaloid metabolites were shared across all four studied *H. micrantha* varieties (Fig. 1a), while no metabolites exclusive to a single variety. 2-(D-Glucosyloxy)-4-hydroxybenzeneacetonitrile was only identified in HT and JQ, while 4,6-Dihydroxyquinoline for YH and HT. Three alkaloid metabolites (4-aminoindole, diethanolamine, and 1-methylhistamine) were absent in HT but present in the other three varieties.

In this study, eight distinct classes, including alkaloids, benzylphenylethylamine alkaloids, phenolamine, piperidine alkaloids, plumerane, pyridine alkaloids, pyrrole alkaloids, and quinoline alkaloids were totally discerned among all the identified alkaloid metabolites (Fig. 1b). Therein, the alkaloid class has the highest number of metabolites, and it covered 45% of total alkaloid metabolites. A total of 21 alkaloid metabolites was detected across four varieties, while only a few of metabolites was identified in other classes.

### Principal component analysis

**Fig. 2:**
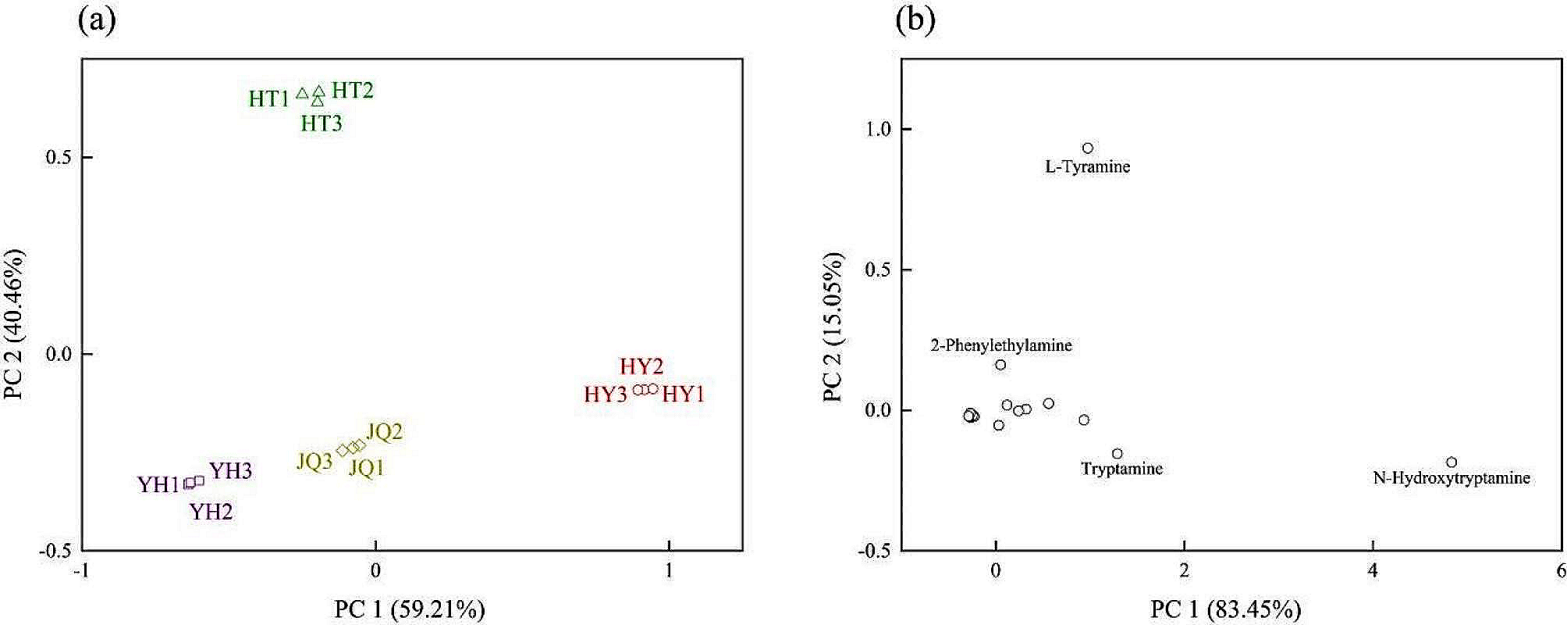
Scatterplot derived of four *H. micrantha* varieties from the principal component analysis based on the alkaloid metabolites (**a**). Scatterplot derived of all alkaloid metabolites from the principal component analysis based on the four *H. micrantha* varieties (**b**). YH, Yongheng; HY, Hongyue; HT, Huatan; JQ, Jinqiu

The results of principal component analysis (PCA) showed that PC 1 and PC 2 accounted for 99.67% of the total variables (Fig. 2a). Therein, PC 1 accounted for 59.21% of the total variables, while 40.46% for PC2. Moreover. the scatter plot shows that the 4 varieties are clearly divided into 4 distinct groups, but the 3 replicates of each variety are closely clustered together. This further validates the reliability of our findings. HY and HT were distinctively separated based on their respective high PC 1 and PC 2 values. While the PC 2 value for YH and JQ was relatively low, they exhibited a noticeable difference in PC 1, resulting in their separation.

The study on the alkaloid metabolites exhibited that PC 1 and PC 2 accumulatively explained 98.50% of the total variables (Fig. 2b). Moreover, PC 1 and PC 2 separately explained 83.45% and 15.05% of the total variables. In addition, N-hydroxytryptamine and tryptamine contributed greatly to PC1, while L-Tyramine for PC2. Furthermore, they exhibited high eigenvalues, 0.92 and 0.96, respectively.

Among them, four varieties all contributed to PC 1, HY contributed the most, and HT contributed the most to PC 2. The scatter plot shows that 44 alkaloid metabolites were divided into several parts, N-hydroxytryptamine was significantly separated due to high PC 1, followed by tryptamine, indicated that these two metabolites were important alkaloid metabolites in the leaves of four varieties. L-Tyramine and 2-phenylethylamine, significantly separated due to their high PC 2 scores, are present in high concentrations specifically in HT variety.

### Analysis of differential alkaloid metabolites

**Fig. 3:**
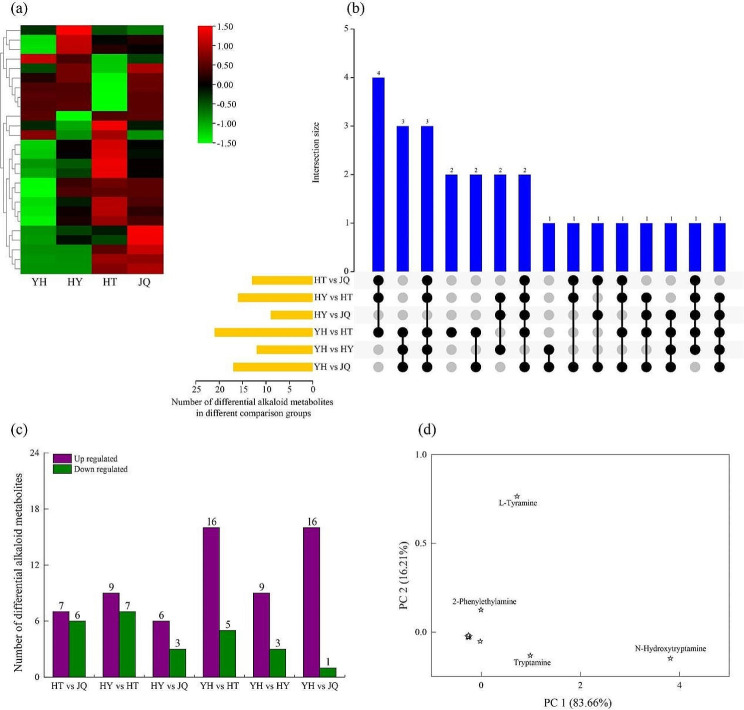
Heatmap of differential alkaloid metabolites of four different *H. micrantha* varieties (**a**). UpSet plot illustrating the overlapping and specific alkaloid metabolites of each comparison group (**b**). Yellow bars represent the total number of alkaloid metabolites isolated from each comparison group. Joined black dots represent intersections between the comparison groups and blue bars represent the number of metabolites common to the marked comparison groups. Up- and down-regulated differential alkaloid metabolites in each comparison group (**c**). Scatterplot derived of differential alkaloid metabolites from the principal component analysis based on the four *H. micrantha* varieties (**d**). YH, Yongheng; HY, Hongyue; HT, Huatan; JQ, Jinqiu

Among the four varieties, a total of 26 out of the 44 identified alkaloid metabolites were different in one or more comparison groups among the four varieties (Fig. 3a). There were 1 ~ 4 differential alkaloid metabolites with differences in 1 ~ 5 comparison groups, while no common differentially alkaloid metabolites was found among all 6 comparison groups (Fig. 3b). Among the 6 comparison groups, only one group (YH vs. HT) had specific differential alkaloid metabolites (indole-3-carboxaldehyde and DL-2-aminoadipic acid). In contrast, there were higher levels of overlapping alkaloid differential metabolites across the other five comparison groups.

Among the 6 comparison groups, 13, 16, 9, 21, 12, and 17 differential alkaloid metabolites were totally identified from HY vs. JQ, HY vs. HT, HY vs. JQ, YH vs. HT, YH vs. HY and YH vs. JQ, respectively (Fig. 3b, c). All these differential alkaloid metabolites include both up- and down-regulated one. Therein, 16 up-regulated differential alkaloid metabolites was found in both YH vs. HT and YH vs. JQ comparison groups (Fig. 3c). While, only 5 and 3 down-regulated differential alkaloid metabolites were separately found in these two comparison groups.

The PCA analysis on differential alkaloid metabolites indicated that PC 1 and PC 2 accounted for 99.87% of the total variables as shown in Fig. 3d. Therein, PC 1 and PC2 accounted for 83.66% and 16.21% of the total variables, respectively. Moreover, N-hydroxytryptamine contributed greatly to PC1, while L-tyramine to PC2.

### K-means cluster analysis of differential alkaloid metabolites

**Fig. 4:**
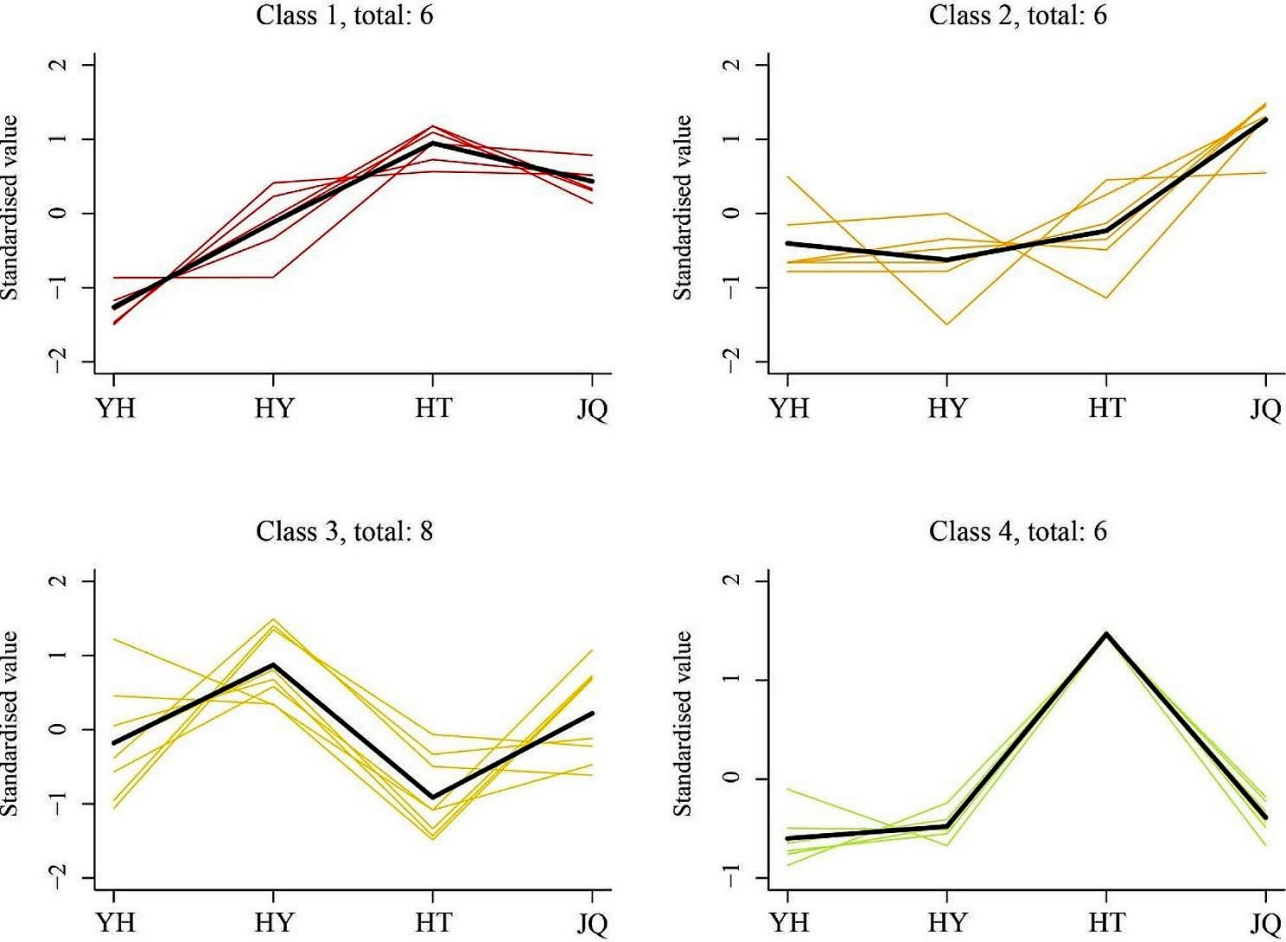
K-means cluster trend map of differential alkaloid metabolites. YH, Yongheng; HY, Hongyue; HT, Huatan; JQ, Jinqiu

In this study, four distinct trends were observed in the alkaloid metabolites profiles based on K-means cluster analysis among the four *H. micrantha* varieties (Fig. 4). Class 1 comprised a total of 6 differential alkaloid metabolites. These metabolites exhibited a higher content in HT varieties, but a lower content in YH varieties. Class 2 and 4 characterized by a higher content in JQ and HT, respectively. Both these two classes encompassed 6 differential alkaloid metabolites. A total of 8 differential alkaloid metabolites was classified into class 3. Moreover, they showed different trends with other three classes. Both HY and JQ have higher content, but lower content in YH and HT.

### Enrichment analysis of differential alkaloid metabolites

**Fig. 5:**
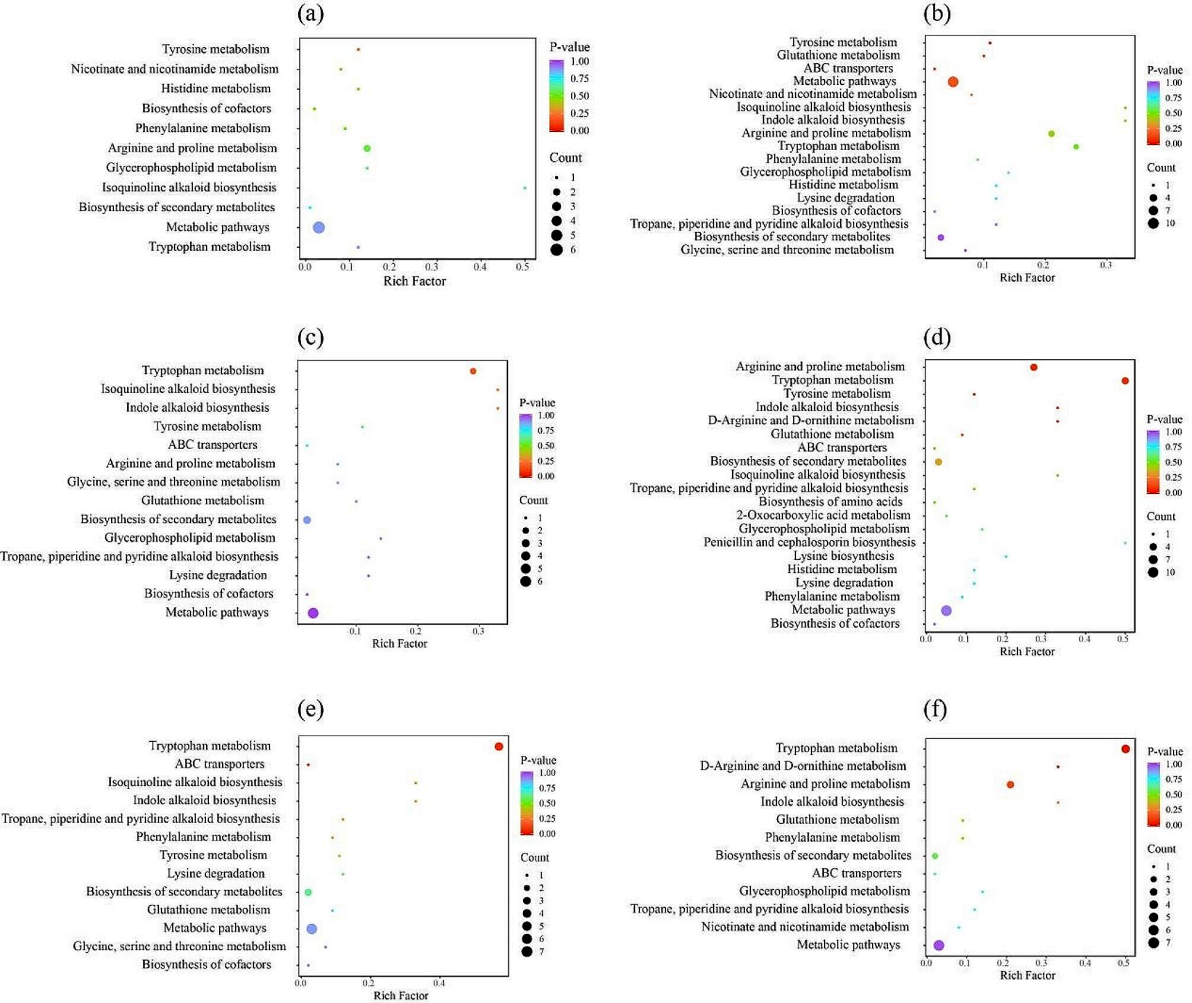
KEGG enrichment of differential alkaloid metabolites for each comparison group, HT vs. JQ (**a**), HY vs. HT (**b**), HY vs. JQ (**c**), YH vs. HT (**d**), YH vs. HY (**e**), YH vs. JQ (**f**). YH, Yongheng; HY, Hongyue; HT, Huatan; JQ, Jinqiu

Analysis of KEGG enrichment for differential alkaloid metabolites across the six comparison groups revealed that 26 differential alkaloid metabolites were enriched into 22 KEGG pathways (Fig. 5). Such as: tyrosine metabolism (ko00350), phenylalanine metabolism (ko00360), tryptophan metabolism (ko00380), indole alkaloid biosynthesis (ko00901), isoquinoline alkaloid biosynthesis (ko00950), tropane, piperidine and pyridine alkaloid biosynthesis (ko00960), and etc. The differential alkaloid metabolites in YH vs. HT comparison group were enriched with the most KEGG pathways (20), while the least KEGG pathways for HT vs. JQ comparison group (11).

Among the 6 comparison groups, the differential alkaloid metabolites were enriched in three common KEGG pathways, including tryptophan metabolism, biosynthesis of secondary metabolites, and metabolic pathways. In most of the KEGG pathways, there was a single differential alkaloid metabolite enrichment. Therein, four KEGG pathways (lysine biosynthesis, biosynthesis of amino acids, penicillin and cephalosporin biosynthesis and 2-oxocarboxylic acid metabolism) were uniquely enriched in YH vs. HT comparison group. None of the other five comparison groups exhibited a unique KEGG pathway enrichment.

### Analysis of differentially expressed genes

**Fig. 6:**
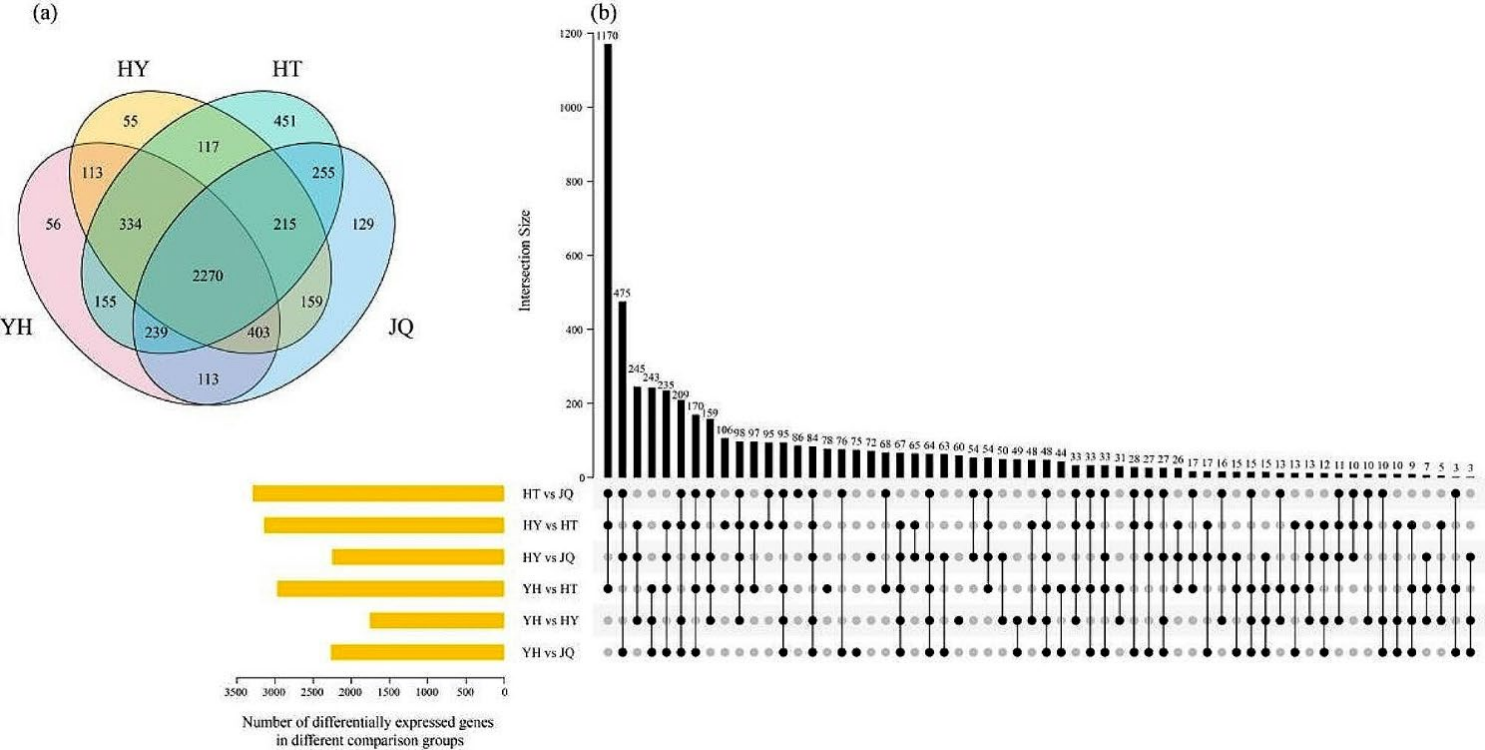
Venn diagram of differentially expressed genes in each of the four *H. micrantha* varieties (**a**). UpSet plot illustrating the overlapping and specific of differentially expressed genes of each comparison group (**b**). Yellow bars represent the total number of differentially expressed genes isolated from each comparison group. Joined black dots represent intersections between the comparison groups and black bars represent the number of differentially expressed genes common to the marked comparison groups

In this study, we totally identified 5064 differentially expressed genes combined with transcriptome data and the previous KEGG pathway results (Fig. 6a). Among these differentially expressed genes, 2270 genes were expressed across all four varieties. However, 56, 55, 41, and 129 genes were uniquely expressed unique in YH, HY, HY, and JQ, respectively.

In addition, there were 3 ~ 1170 differentially expressed genes with differences in 1 ~ 6 comparison groups (Fig. 6b). Each comparison group harbored distinct genes. The highest number of overlapping genes (1170) was observed in three comparison groups, including HT vs. JQ, HY vs. HT, and YH vs. HT. Moreover, these three comparison groups also showed the highest differentially expressed genes.

**Fig. 7:**
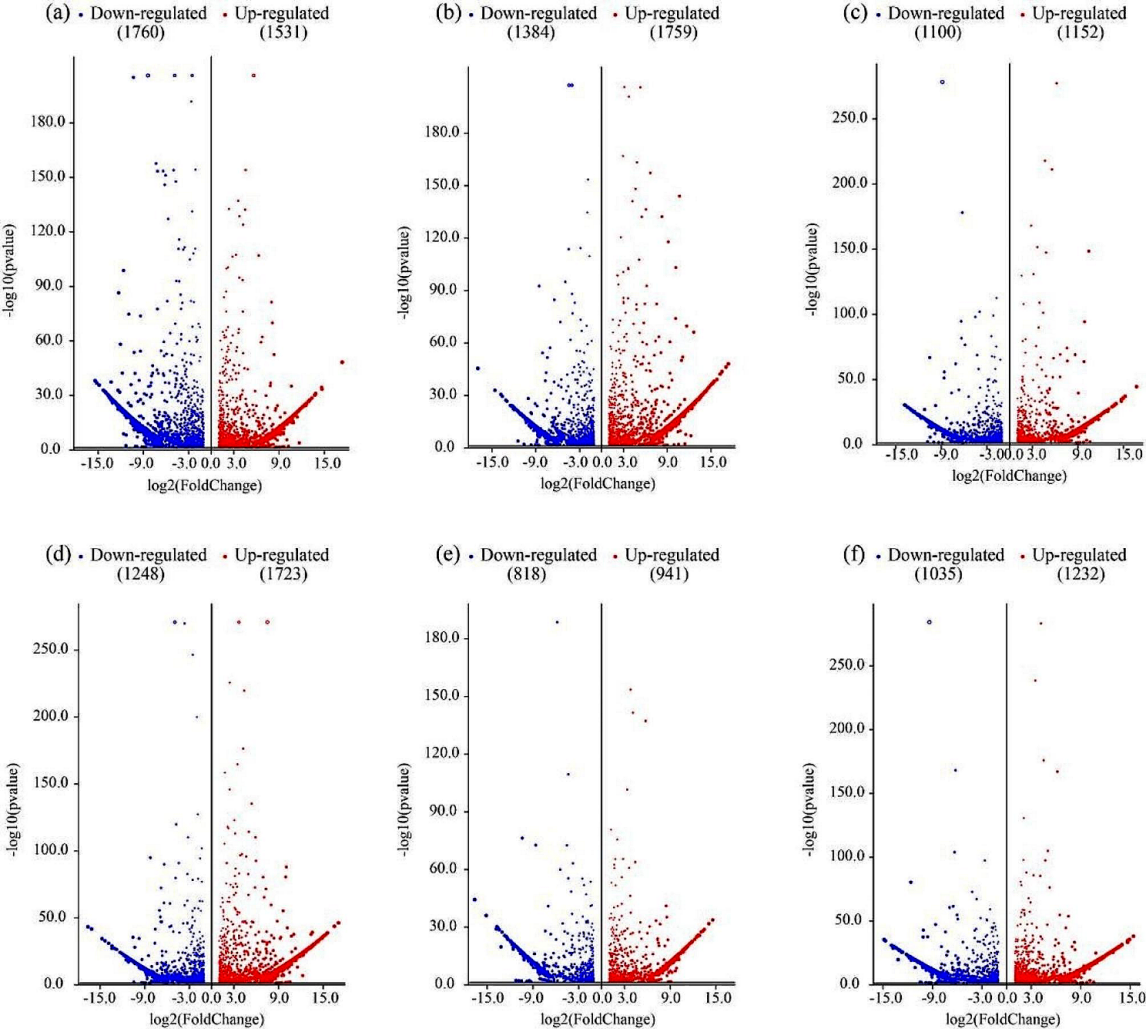
Volcano map of differentially expressed genes for each comparison group, (**a**) HT vs. JQ, (**b**) HY vs. HT, (**c**) HY vs. JQ, (**d**) YH vs. HT, (**e**) YH vs. HY, (**f**) YH vs. JQ. YH, Yongheng; HY, Hongyue; HT, Huatan; JQ, Jinqiu

A total of 48 overlapping genes were differentially expressed across all these six comparison groups (Fig. 7). In this study, both HT vs. JQ (3291) and HY vs. HT (3143) comparison groups showed the highest differentially expressed genes, 3291 and 3143, respectively (Fig. 7a, b). In HT vs. JQ comparison groups, it contained 1531 up-regulated and 1760 down-regulated. In HY vs. HT comparison groups, the up-regulated differentially expressed genes is 1759, 1384 for down-regulated one. However, YH vs. HY comparison group has the lowest one (1759; Fig. 7e). Moreover, there was only 941 up-regulated differentially expressed genes, and 818 down-regulated ones.

### K-means cluster analysis of differentially expressed genes

**Fig. 8:**
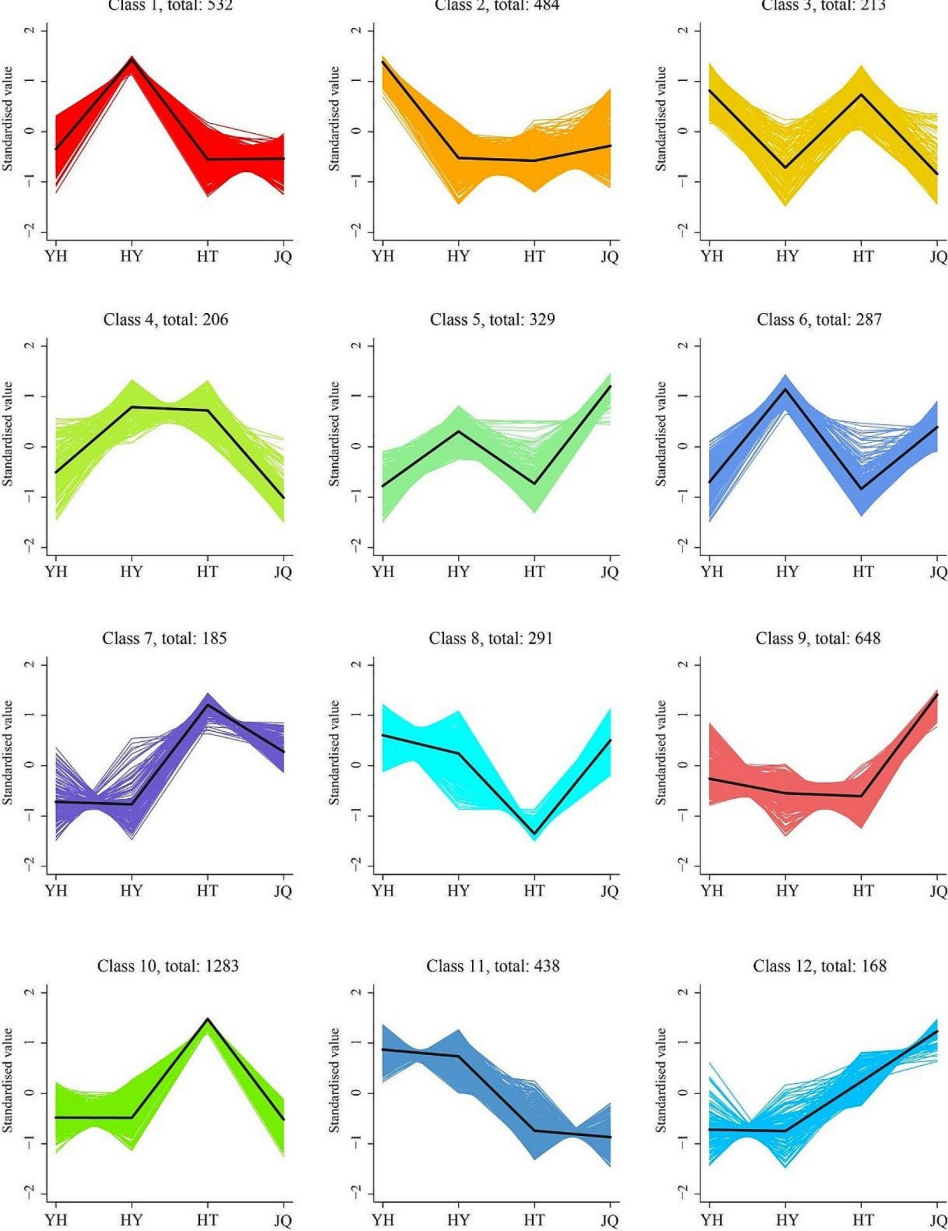
K-means cluster trend map of differentially expressed genes. YH, Yongheng; HY, Hongyue; HT, Huatan; JQ, Jinqiu

The gene exhibiting differentially expressed were categorized into 12 distinct Classes (Class 1 ~ Class 12) as depicted in Fig. 8. Class 12 included the largest number of differentially expressed genes (1283), classes 9 and 1 were secondary. The class 12 contains the fewest differentially expressed genes, but also 168. Among the 12 classes, the expression of differentially expressed genes showed different patterns in the studied four varieties. Variety YH has the largest number of differentially expressed genes in classes 2, 3, 8, and 11. Variety HY has the largest in classes 1,4, and 6. Variety HT has the largest in classes 3, 7, and 10. While, variety JQ has the largest number in classes 5, 8, 9, and 12.

### Correlation analysis

**Fig. 9:**
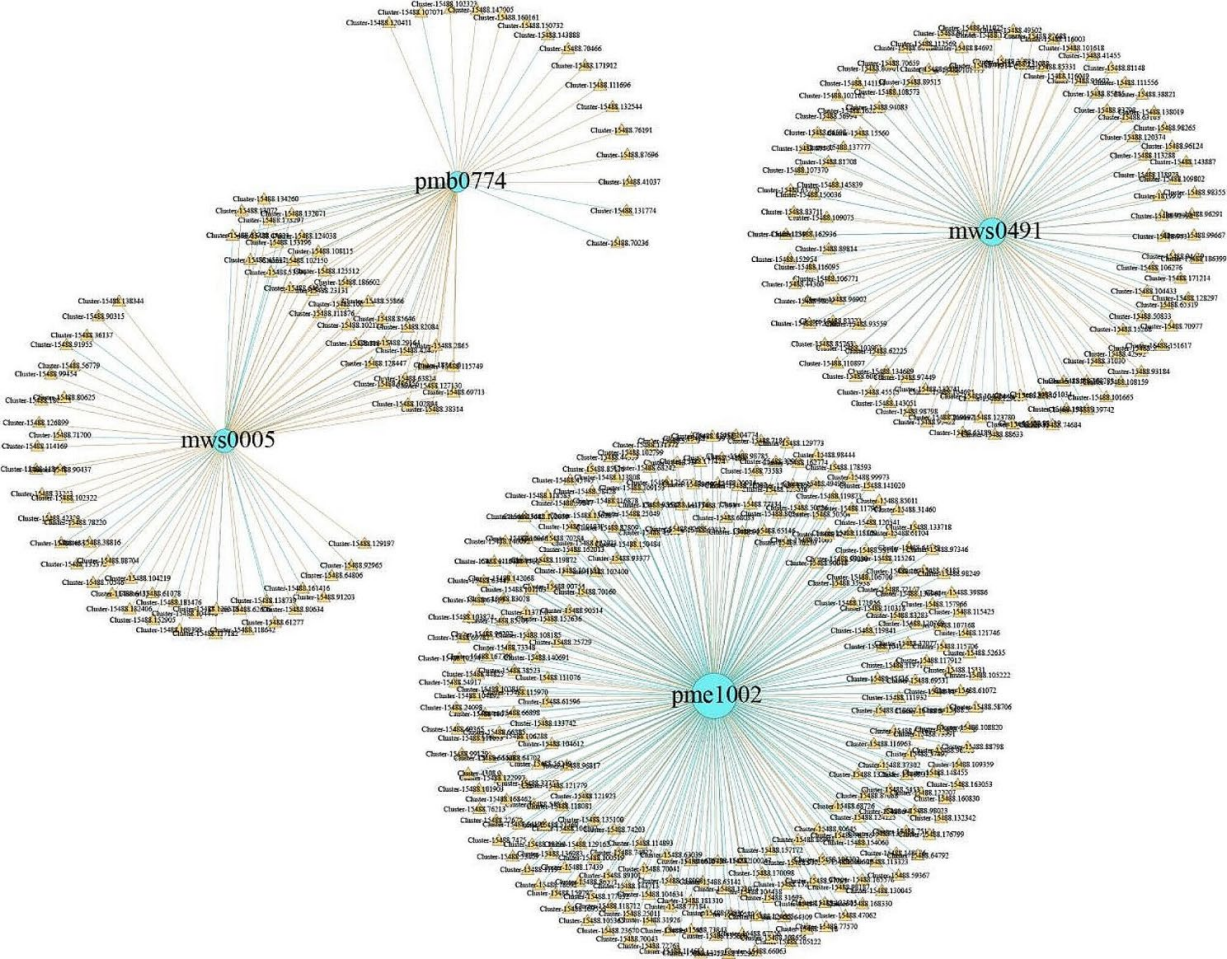
Regulatory network map of differential alkaloid metabolites and differentially expressed genes. Blue circles indicate differential alkaloid metabolites, yellow triangles indicate differentially expressed genes, blue lines indicate negative regulation, and yellow lines indicate positive regulation. Mws0005, tryptamine; Mws0491, 2-phenylethylamine; Pmb0774, N-hydroxytryptamine; Pme1002, L-tyramine

To further identified candidate genes differentially expressed and regulating key alkaloid metabolites in *H. micrantha* leaves, the Pearson method was used to analyze the association between the two omics datasets (Fig. 9). Among the differentially expressed genes, the highest correlation was observed with L-tyramine (pme1002), totaling 277 genes, of which 197 were negatively correlated and 80 were being positively correlated. The 2-phenylethylamine (mws0491) were significantly correlated with 116 genes, including 55 negatively correlated genes and 61 positively correlated genes. Additionaaly, 77 genes were significantly correlated with tryptamine (mws0005), while 51 genes were significantly correlated with N-hydroxytryptamine (pmb0774). Furthermore, 35 genes were found to be significantly correlated with both these two metabolites.

### Analysis of candidate genes

**Fig. 10:**
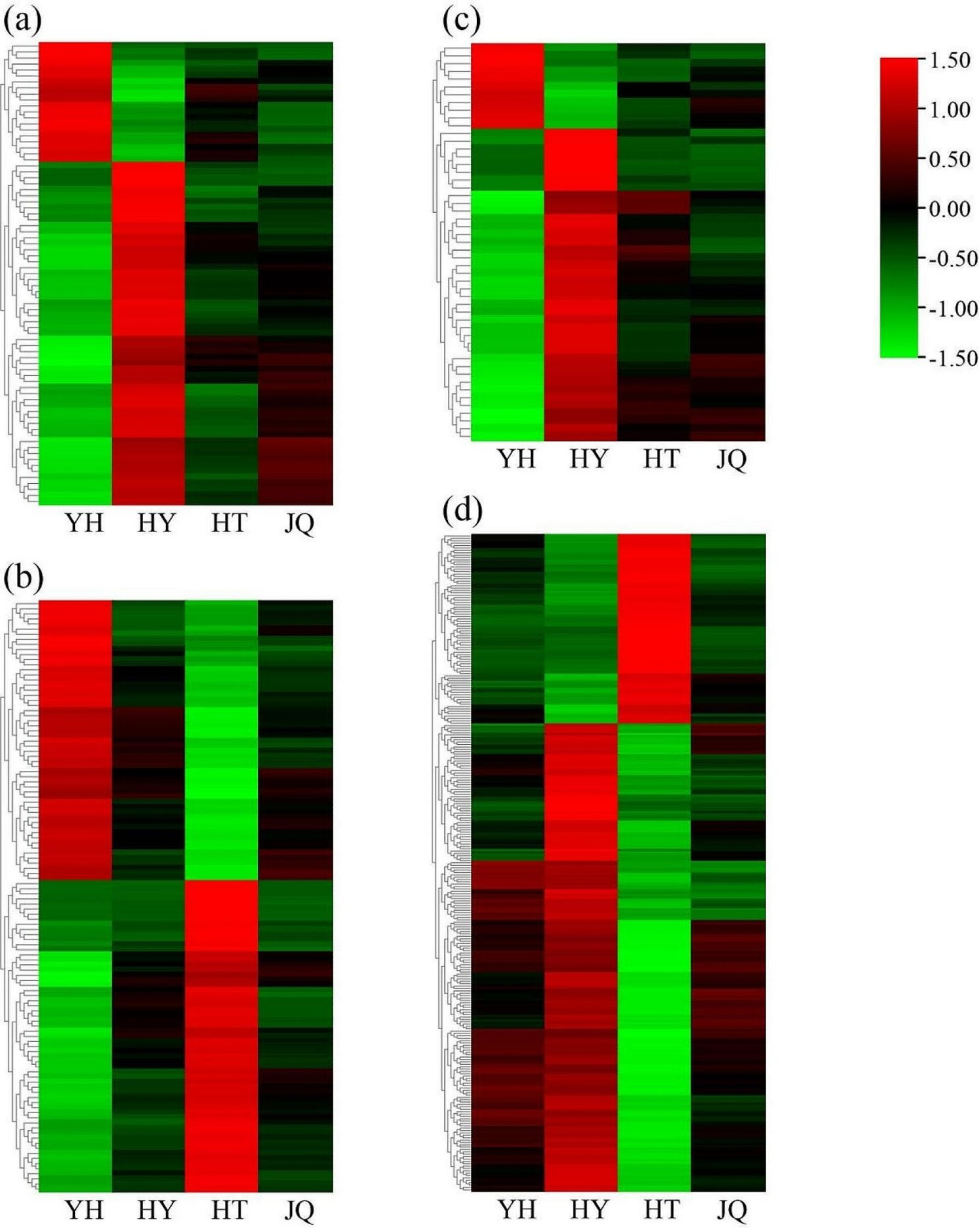
Heatmap of differentially expressed genes with differential alkaloid metabolites, (**a**) tryptamine, (**b**) 2-phenylethylamine, (**c**) N-hydroxytryptamine, (**d**) L-tyramine; YH, Yongheng; HY, Hongyue; HT, Huatan; JQ, Jinqiu

The expression patterns of the differentially expressed genes screened by correlation analysis in the leaves of four *H. micrantha* varieties were plotted through heat map (Fig. 10). A total of 20 genes, exhibiting significant association with tryptamine were highly expressed in YH compared to the other three varieties. Compared to the other three varieties, HY exhibited higher expression of 57 differentially expressed genes (Fig. 10a). The differentially expressed genes markedly correlated with N-hydroxytryptamine showed the same trend (Fig. 10c). And a total of 40 differentially expressed genes was identified. Eleven differentially expressed genes were highly expressed in YH compared to the other three varieties (Fig. 10c). The differentially expressed genes significantly correlated with 2-phenylethylamine were highly expressed in YH and HT (Fig. 10b), while the genes significantly correlated with L-tyramine were primarily expressed in HY and HT (Fig. 10d).

### Analysis of alkaloid metabolic network

**Fig. 11:**
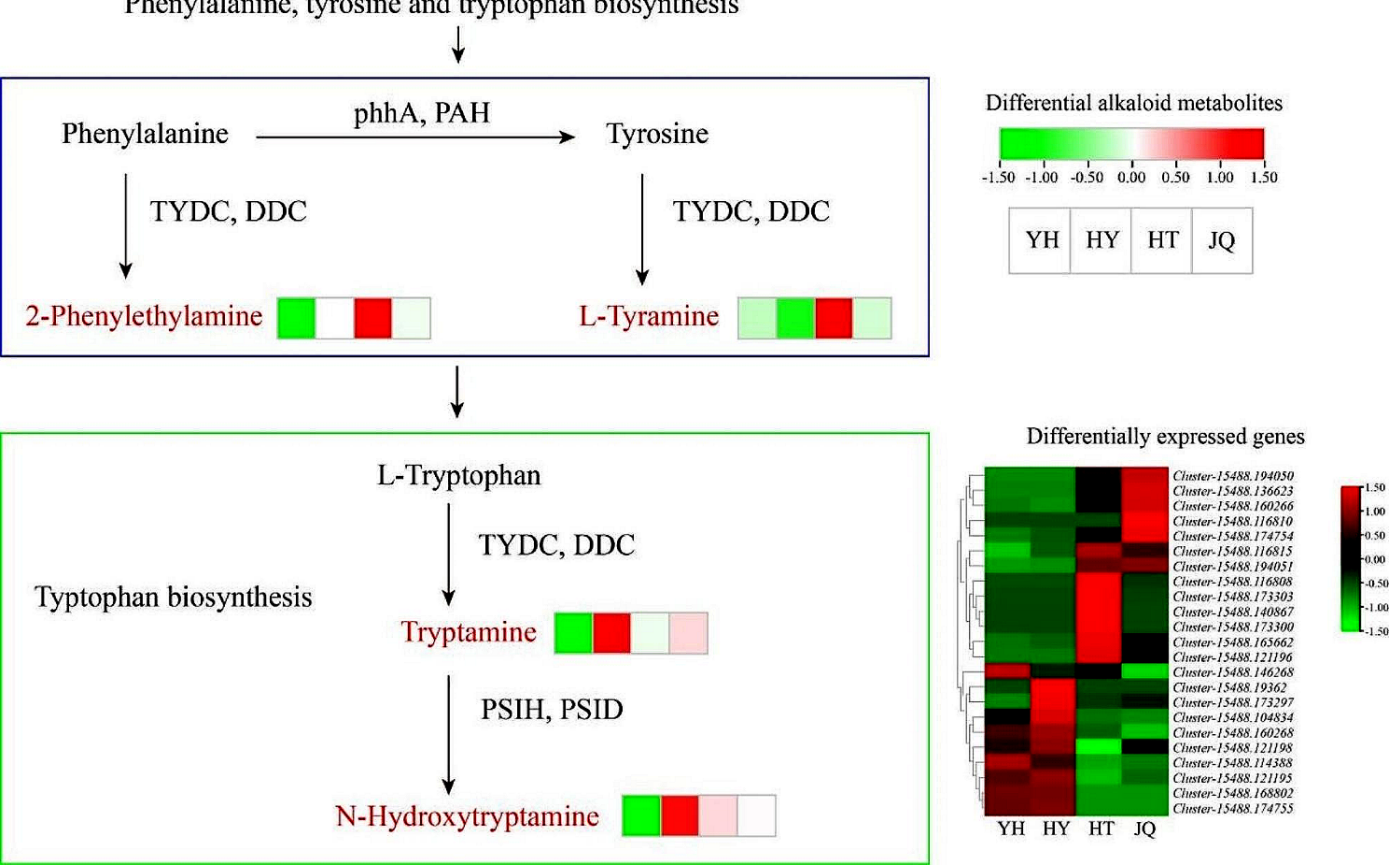
Network of alkaloid metabolism. YH, Yongheng; HY, Hongyue; HT, Huatan; JQ, Jinqiu

L-tyramine and 2-phenylethylamine are enriched in phenylalanine, tyrosine and tryptophan biosynthesis (Fig. 11). Both of these metabolites exhibit the highest content in HT. 2-phenylethylamine is synthesized by phenylalanine in the presence of aromatic-L-amino-acid (TYDC, DDC), and L-tyramine is synthesized by phenylalanine in the presence of tyrosine in the presence of phenylalanine hydroxylase (phhA, PAH) and then in the presence of TYDC. Tryptamine and N-hydroxytryptamine were found to be enriched in typtophan biosynthesis (Fig. 11), and these two metabolites both have the highest content in HY. Under the catalysis of TYDC, L-tryptophan produces tryptamine, which is then converted to N-hydroxytryptamine by tryptamine 4-monooxygenase (PSIH, PSID). Following further screening based on metabolic network, a total of 23 genes related to the four improtant metabolites were identified and their expression levels were heat-mapped (Fig. 11). Analysis of gene expression levels and trends led to the identification of three candidate genes: *Cluster-15488.116815*, *Cluster-15488.146268*, and *Cluster-15488.173297*.

## Discussion

Alkaloid metabolites, a subclass of secondary metabolites, are ubiquitous in plant species. Alkaloid metabolites exhibit anti-tumor, anti-bacterial, anti-viral and analgesic activities, making them a crucial component of traditional Chinese medicine. Conseuencently, recent research has centered on alkaloid metabolites, particularly their identification and pharmacological effects in traditional Chinese medicinal herbs and edible plants. A total of 18 alkaloids were identified to be highly concentrated in the root of *Veratrum mengtzeanum*, including the novel alkaloid compound 3-Vanilloylygadenine. Due to its unique alkaloid components, it has become the main component of Chinese folk medicine [14]. The administration of Veratrum alkaloid resulted in a rapid decreased in BP within 30 min, attenuation of renal and cardiovascular damage, and improvement of biochemical indicators such as nitric oxide [NO], endothelin-1 [ET-1], angiotensin II [Ang II)], noradrenaline [NE], etc.) in SHRs, thereby delaying the occurrence of stroke [15]. As a significant component of traditional Chinese medicine, *Coptis chinensis* has been metabolomically analyzed to reveal a total of 413 alkaloids [16]. The isoquinoline alkaloid is the main active component of *C. chinensis*, mainly exists in its rhizomes and has high clinical application potential. *Ziziphus spinosa*, as a medicinal and edible plant, has also identified multiple alkaloid metabolites at different developmental stages [17]. *Phellodendron amurense* (Rupr.) is a well-known medicinal plant with high medicinal value, and its various tissues are enriched in various active pharmaceutical ingredients. Isoquinoline alkaloids are the primary medicinal component of *P. amurense* and have multiple effects, such as anti-inflammation, antihypertension, and antitumor effects [18].

Although alkaloids exhibit potential biological activity, recent studies increasingly demonstrate their toxic properties. *Eupatorium fortune*, a traditional Chinese herbal medicine, has found widespread use in treating nausea, diabetes, siriasis, and poor appetite. Notably, *E. fortunei* contains a diverse array of pyrrolizidine alkaloids. The pyrrolizidine alkaloids induced severe hepatotoxicity by disrupting glycerophospholipid metabolism [12]. Alkaloids, a class of secondary metabolites, are produced by plants in response to habitat stress, according to Zhu et al. (2023) [19]. There is a complex relationship between alkaloid metabolites and biotic and abiotic stresses [9]. These stresses may come from biotic factors, such as insects, pathogens, etc., may also come from abiotic factors, such as extreme temperature, drought, salinity, etc. [20]. Under such stresses, plants synthesize and accumulate alkaloids to enhance their defenses. Additionaly, the bitter taste of many alkaloids serves as a deterrent to animals, preventing grazing and thus serving a protective purpose, as demonstrated by Otterbach et al. (2019) [21]. Additionally, certain alkaloids exhibit anti-bacterial function, aiding plants in defending against microorganisms, as reported by Melander et al. (2016) [22]. furthermore, alkaloids play a role in plants’ ability to tolerate environmental stress. Plants respond to stress by adjusting alkaloid synthesis and metabolism. For instance, some plants under drought stress, will increase the production of alkaloids to improve their drought tolerance [23]. Lastly, alkaloids are involved in regulating endogenous metabolism-related pathways [24]. These pathways include hormone synthesis, signal transduction and energy metabolism, which are closely related to plant stress response and adaptation. Consequently, alkaloid metabolites play an important role in plants’ response to both biotic and abiotic stresses. These metabolisms not only contribute to plant defense mechanisms, but also influence plant physiological metabolism and adaptability. In this study, four distinct ecological types of *H. micrantha* varieties (Yongheng, Hongyue, Huatan, and Jinqiu) were selected as experimental materials, and their alkaloid metabolites were determined and analyzed. Forty-four alkaloid metabolites were detected in the leaves of four varieties of *H. micrantha*. Classification of the identified alkaloid metabolites revealed eight distinct classes, and these classes encompassed alkaloids, benzylphenylethylamine alkaloids, phenolamine, piperidine alkaloids, plumerane, pyridine alkaloids, pyrrole alkaloids, and quinoline alkaloids. Analysis of differential alkaloids metabolites identified a total of 26 differential alkaloids metabolites, and they were primarily accumulated in HT and JQ than in YH and HY. Additionally, four important metabolites (N-hydroxytryptamine, L-tyramine, tryptamine, and 2-phenylethylamine) were selected for further analysis.

Alkaloids, serving as chemical defense substances, play an important role in plant resistance to biotic stress and have always been an important source of drug development for humans [25]. The diverse origins of alkaloid precursors and the intricate transformations of the ring system during skeleton formation leads to the loss of structural information of primary metabolic components in the final alkaloid product. Consequently, determining the biosynthetic source and structural alterations becomes challenging, significantly elevating the complexity of analyzing alkaloid biosynthesis pathways [26]. Therefore, it is necessary to break the biosynthesis mechanism of alkaloids to realize the efficient and high-quality production of related drugs by using the green platform in the future. The research of Chinese jujube and sour jujube identifiedkey metabolites and 11 candidate genes through mapping the regulatory networks of differential alkaloid metabolites and differentially expressed genes [18]. We mined potential CYP450 gene from the cDNA library of the celestial fairy. Utilizing VIGS (virusinducedgenesilencing) technology found that one of the candidate genes-CYP80F1 expression after the downgrade, scopolamine content in plant body drops, conch alkali content rise. The function was subsequently confirmed through enzyme reactions in tobacco and P450 microsomes in vitro [27]. The researchers utilized the *Atropa belladonna* transcriptome to screen for *HDH* candidate genes that are specifically highly expressed in lateral root tissues. By conducting in vitro enzymatic reactions, protein crystal structure analysis, and deuterium atom labeling experiments, they found that HDH is an oxidoreductase that can catalyze the bidirectional conversion of alkaloids [28]. The identification and functional characterization of HDH is a representative idea and method for mining and analyzing the key enzyme genes of natural product biosynthesis. Candidate genes potentially catalyzing these two steps were screened from the transcriptome of *Catharanthus roseus*, and the corresponding oxidases *PAS* and *DPAS* were found. Transient expression of *PAS*, *DPAS* and *CS* or *TS* in tobacco, while providing acetyl corolla lye, successfully detected the formation of catharanthine and its bonin in tobacco leaves. In an in vitro one-pot enzyme reaction, the identical conclusion was researched through the utilization of CS, TS and DPAS expressed in *E. coli*, along with PAS expressed in tobacco, as reported by Caputi et al. (2018) [29]. The above experimental design ideas provide an important reference for the analysis of intermediate products that are not yet clear. In this study, the regulatory networks of four important differential metabolites and differentially expressed genes were constructed by combined analysis of metabolomic and transcriptomic. Multiple regulatory genes were found for each differential metabolite, and the metabolic pathways of differential metabolites and differentially expressed genes were analyzed. A total of three differentially expressed genes (*Cluster-15488.116815*, *Cluster-15488.146268*, and *Cluster-15488.173297*.) were chosen as candidate genes related to the metabolism of *H. micrantha* which were all *TYDC* (L-tryptophan decarboxylase) genes. *TYDC* is located in the initial position of benzyl isoquinoline alkaloids (BIAs) biosynthesis, which plays an important role in the process of BIAs synthesis. In *Macleaya cordata*, *McTYDC2* gene is mainly expressed in the roots of *M. cordata*, and the root tissue of *M. cordata* is considered to be the main site of BIAs synthesis. Therefore, the expression rule of this gene is consistent with the metabolism rule of *M. cordata* alkaloid [30]. By transferring the *TYDC* gene in opium poppy into canola, the level of cell wall-bound tyramine was increased and the cell wall digestibility was reduced [31]. Through expression analysis and sequence analysis, two *TYDC* genes were screened to be related to the betalain biosynthesis pathway of pitaya, which may play a role in the betalain biosynthesis pathway [32].

In the future research, the function of candidate genes can be verified, the regulatory relationship between genes and important alkaloid metabolites can be clarified, and the gene-metabolite-ecotype can be further combined with the habitat type of *H. micrantha*, so as to provide an important basis for breeding *H. micrantha* varieties with strong adaptability.

## Conclusions

Our study conducted a thorough and methodical assessment of alkaloid metabolites accumulation across four distinct varieties of *H. micrantha*. Based on the metabolome and transcriptome, four important alkaloids metabolites and three candidate genes were selected and preliminarily expounded the biosynthetic pathway of alkaloid metabolites. Nevertheless, the diverse environmental ecotype of these four varieties remains unexplored. The results of this study will provide important help for directional breeding and molecular breeding of alkaloids metabolites with high content in future work. At the same time, it can provide references for landscape selection of varieties with high resistance, and also provide a basis for the development of medicinal value of *H. micrantha*.

## Materials and methods

### Plant materials

Four varieties (Yongheng, Hongyue, Huatan, and Jinqiu) of *Heuchera micrantha* Douglas ex Lindl. were cultivated in the greenhouse at Honghe University (Mengzi, Yunnan, China). All seedlings were obtained from Lvmeng flower company. For each variety, a total of nine healthy plants were chosen, with each plant replicated three times, resulting in three biological repetitions per variety. The sampled leaves were stored at -80 °C prior to analysis.

### Sample preparation and extraction

To prepare the leaf samples for UPLC-MS/MS analysis, leaf samples were placed in a lyophilizer (Scientz-100 F) and vacuum freeze-dried. The freeze-dried leaves were then ground using an MM 400 pulverizer (Retsch GmbH, Haan, Germany) [33] for 1.5 min at 35 Hz. Next, 100 mg of the leaf powder was weighed and dissolved in 1.2 mL of 70% methanol solution for vortex extraction. Each sample was vortexted 6 times for 30 s, and was then left to stand for 30 min. The processed samples were then placed overnight at 4 °C. The samples were then centrifuged at 12,000 × g for 10 min, after which 1.0 mL of the supernatant was passed through a 0.22 μm Millipore membrane filter and placed in a liquid sample bottle for UPLC-MS/MS analysis.

### Qualitative and quantitative metabolite analysis

Metabolites were analyzed using ultra performance liquid chromatography (UPLC) and tandem mass spectrometry (MS/MS). The determination of metabolite identities was carried out through analysis of the precursor ion (Q1), product ion (Q3), retention time (RT), fragmentation mode and molecular weight with injection standard under the same conditions. Based on the self-built Metware database, the qualitative analysis was carried out using the second-order spectrum information, and for the quantitative analysis of metabolites, we employed a multiple reaction monitoring (MRM) method using the triple-quadrupole mass spectrometry.

The conditions for liquid chromatography were as follows. The column was an Agilent SB-C18 column (2.1 mm × 100 mm; 1.8 μm). The solvent system consisted of 0.1% (v/v) formic acid water (solvent A) and acetonitrile with 0.1% (v/v) formic acid (solvent B). The flow rate was set to 0.35 mL·min^− 1^, the sample injection volume was 4 µL, and the column temperature was 40 °C. The gradient program was set as follows: 5% B at 0 min, with the proportion of B increasing linearly to 95% within 9 min and remaining there for 1 min, after which the proportion of B was decreased to 5% between the 10th and the 11th minute. The program was equilibrated to 14 min.

The mass spectrum conditions were as follows. LIT and QQQ scans were obtained on an Q TRAP (AB4500 Q TRAP UPLC/MS/MS) system equipped with ESI Turbo ion spray interface, which can run both positive and negative ion modes under the control of the Analyst 1.6.3 software. ESI source operating parameters were as follows. Ion Source, turbine spray; source temperature 550 °C; ion spray voltage 5500 V (positive mode)/-4500 V (negative mode); ion source gas I (GSI), gas II (GSII) and gas curtain gas (CUR) were set to 50, 60 and 25.0 psi, respectively, and the parameters of collision-induced ionization were set to high. In QQQ and LIT modes, 10 and 100 µmol/l peg solution were used for tuning and quality calibration. QQQ scans using MRM mode and sets collision gas (nitrogen) to medium. Through further optimization of DP and CE, the DP and CE of each MRM ion pair were achieved. A specific set of MRM ion pairs was monitored during each period, based on the metabolites eluted during each period.

### RNA sequencing

The RNA-seq sequencing and assembly were performed by Metware Biotechnology Co., Ltd. (Wuhan, China). Total RNA was extracted from leaves of JQ, HT, HY, and JQ. The integrity of RNA and the presence of DNA contamination were analyzed by agarose gel electrophoresis, the purity of RNA was assessed using a Nanophotometer, the concentration of RNA was measured using Qubit 2.0 fluorescent reagent, and the RNA integrity was assessed using an Agilent 2100 bioanalyzer. After the library was certified, the different libraries were pooled according to the target amount of off-line data and sequenced on the Illumina HiSeq platform.

For RNA-seq, a differentially expression analysis of the four varieties was performed using the DESeq2 method [34, 35]. After the difference analysis, the hypothesis test probability (*p*-value) was corrected for multiple hypothesis testing to obtain the false discovery rate (FDR) using the Benjamini-Hochberg method. The screening conditions for DEGs was |log2foldchange| ≥ 1 and FDR < 0.05.

### Statistical analysis

To compare alkaloid metabolite profiles among four varieties of *H. micrantha*, metabolites exhibiting a fold change ≥ 2 or fold change ≤ 0.5 were selected, and metabolites with VIP ≥ 1 were selected at the same time.

The data were analyzed using Microsoft Excel v. 2007 (Microsoft Corporation, Redmond, WA, USA). Principal component analysis (PCA) was performed utilizing the Statistical Analysis System v. 9.2 (SAS Institute, Cary, NC, USA). Tbtools (Chen et al., 2020) and OriginPro9.0 (OriginLab Corporation, Northampton, MA, USA) were used to draw the figures. Pearson correlation analysis was performed to assess the relationship between differential alkaloid metabolites and differentially expressed genes [36]. Values were normalized using log2 transformation, with a threshold of 0.8 for association analysis and a p-value threshold of 0.05 [37]. K-means clustering algorithm used R version 4.2.0 to analyzed [38], the criteria for selecting the number of clusters according to the Calinski-Harabasz algorithm. The volcano plot combines the statistical significance measure (P Value) and the Fold Change in statistical analysis. The FC threshold is 2.0, and the P value threshold is 0.5 [39].

### Electronic supplementary material

Below is the link to the electronic supplementary material.

Supplementary Material 1

## Data Availability

The datasets generated and/or analyzed during the current study are available in the NCBI Sequence Read Archive (SRA) repository, BioProject’s metadata is available at https://dataview.ncbi.nlm.nih.gov/object/PRJNA1105328?reviewer= kb3r9bvdbqneu507h2qm2cmoc2.
